# The outer membrane protein Tp92 of *Treponema pallidum* induces human mononuclear cell death and IL‐8 secretion

**DOI:** 10.1111/jcmm.13879

**Published:** 2018-09-14

**Authors:** Xi Luo, Xiaohong Zhang, Lin Gan, Chenglong Zhou, Tie Zhao, Tiebing Zeng, Shuangquan Liu, Yongjian Xiao, Jian Yu, Feijun Zhao

**Affiliations:** ^1^ Institute of Pathogenic Biology and Key Laboratory of Special Pathogen Prevention and Control of Hunan Province Collaborative Innovation Center for New Molecular Drug Research University of South China Hengyang China; ^2^ Department of Histology and Embryology School of Medicine University of South China Hengyang China; ^3^ Department of Clinical Laboratory The First Affiliated Hospital of University of South China Hengyang China; ^4^ Department of Clinical Laboratory The Second Affiliated Hospital of University of South China Hengyang China

**Keywords:** apoptosis, CD14, IL‐8, membrane protein, pyroptosis, TLR2, Tp92, *Treponema pallidum*

## Abstract

*Treponema pallidum* is the pathogen that causes syphilis, a sexually transmitted disease; however, the pathogenic mechanism of this organism remains unclear. Tp92 is the only *T. pallidum* outer membrane protein that has structural features similar to the outer membrane proteins of other Gram‐negative bacteria, but the exact functions of this protein remain unknown. In the present study, we demonstrated that the recombinant Tp92 protein can induce human mononuclear cell death. Tp92 mediated the human monocytic cell line derived from an acute monicytic leukemia patient (THP‐1) cell death by recognizing CD14 and/or TLR2 on cell surfaces. After the stimulation of THP‐1 cells by the Tp92 protein, Tp92 may induce atypical pyroptosis of THP‐1 cells via the pro‐caspase‐1 pathway. Meanwhile, this protein caused the apoptosis of THP‐1 cells via the receptor‐interacting protein kinase 1/caspase‐8/aspase‐3 pathway. Tp92 reduced the number of monocytes among peripheral blood mononuclear cells. Interestingly, further research showed that Tp92 failed to increase the tumour necrosis factor‐α, interleukin (IL)‐1β, IL‐6, IL‐10, IL‐18 and monocyte chemotactic protein 1 (MCP)‐1 levels but slightly elevated the IL‐8 levels via the Nuclear Factor (NF)‐κB pathway in THP‐1 cells. The data suggest that Tp92 recognizes CD14 and TLR2, transfers the signal to a downstream pathway, and activates NF‐κB to mediate the production of IL‐8. This mechanism may help *T. pallidum* escape recognition and elimination by the host innate immune system.

## 1 INTRODUCTION

Syphilis is a sexually transmitted disease caused by *Treponema pallidum*, affecting millions of people around the world every year.^1^ After an asymptomatic incubation period of 9‐90 days, *T. pallidum* enters blood and lymph circulation from the site of infection, such as local ulcers in the genital mucosa, consequently spreading to all organs and causing the proliferation of systemic chronic inflammatory lesions on the skin and mucosa.^2^ Patients with syphilis who are either not treated at all or are not treated in strict accordance with the prescribed standards may suffer from chronic and persistent infections in the body.^3^ Therefore, *T. pallidum* is likely to have some mechanisms that can affect the immune system, especially mechanisms for evading the innate immune response.

Appropriate killing of innate immune response cells that engulf pathogens would release the pathogens and expose them to the antibacterial machinery of the host; meanwhile, the infected innate immune response cells would be eliminated.^4^

If these important innate immune response cells are eliminated in large quantities, the responsiveness of the host's innate immune response system to early infection will be greatly reduced.^5^ Therefore, via this mechanisms, pathogens may induce the death of a large number of innate immune response cells, thereby evading elimination by the host's immune cells. The regulation of multiple cell‐death‐associated signalling pathways may be involved in pathogenic infection. For example, apoptosis, which depends on receptor‐interacting protein kinase 1 (RIPK1)/caspase‐8/caspase‐3, and pyroptosis, which depends on caspase‐1, are important cell‐death‐associated signalling pathways.^6^, ^7^ Some pathogenic Spirochaeta induce the death of innate immune response cells. For example, *Borrelia burgdorferi*, the pathogenic bacterium responsible for Lyme disease, induces the apoptosis of peripheral blood mononuclear cells (PBMCs).^8^ The leptospiral lipoprotein LIC11207 increases the apoptotic rate of its host neutrophils.^9^ However, to date, there has been no report on whether *T. pallidum* induces the apoptosis of innate immune response cells.

When Gram‐negative bacteria invade hosts, bacterial antigens that are directly exposed to the external environment are the first to interact with the host's innate immune response system. These antigens, such as lipopolysaccharides (LPSs), outer membrane proteins and outer membrane lipoproteins, are instantly recognized by the innate immune response system, leading to a series of immunopathological effects and the activation of immune escape mechanisms.^10^, ^11^ *T. pallidum* lacks the key virulence factor LPS and other common virulence factors, such as exotoxin, that are secreted by other Gram‐negative bacteria.^12^ However, *T. pallidum* can still cause persistent infection and immune damage in patients who have not been treated at all or as prescribed.^3^ It is believed that the outer membrane proteins and lipoproteins of *T. pallidum* play key roles. There are seven variable regions in the open reading frame of the outer membrane protein TprK of *T. pallidum*, and the antigenic variability of this protein may be an immune escape mechanism of *T. pallidum*.^13^ However, there have been no reports on whether other outer membrane proteins or lipoproteins of *T. pallidum* contribute similarly or otherwise to immune escape.

Tp92 is the only *T. pallidum* outer membrane protein that has structural features that are similar to those of the outer membrane proteins of other Gram‐negative bacteria^14^; however, its exact functions of this protein remain unclear. A study showed that the gene encoding the Tp92 protein may be associated with the pathogenesis of *T. pallidum*.^15^ However, the pathogenic effect of Tp92 remains poorly understood. In a recent study, Jun et al demonstrated that Td92, a surface protein of the periodontal pathogen *Treponema denticola* and a homologue of the *T. pallidum* surface protein Tp92, activates caspase‐4 and induces pyroptosis in primary cultured human gingival fibroblasts via cathepsin G activation.^16^ In the present study, we investigated the potential pathogenic role of the outer membrane protein Tp92 by exploring the effect of Tp92 on the THP‐1 innate immune response cells.

## 2 MATERIALS AND METHODS

### 2.1 Chemicals and reagents

Staurosporine (STS, HY‐15141) was purchased from Monmouth Junction (MCE) (NJ, USA). LPS (L2880), peptidoglycan (PGN, 69554) and nigericin (Nig, N7143) were purchased from Sigma‐Aldrich (Darmstadt, Germany). Normal saline was purchased from the Second Affiliated Hospital of University of South China.

The pan‐caspase inhibitor Z‐VAD‐FMK, caspase‐1 inhibitor VX‐765 were purchased from Biovision (Milpitas, CA, USA). The caspase‐3 inhibitor Z‐DEVD‐FMK and caspase‐8 inhibitor Z‐IETD‐FMK were obtained from Biovision. The RIPK1 inhibitor necrostatin‐1 was purchased from Sigma‐Aldrich. The bicinchoninic acid (BCA) assay kit was purchased from Thermo Fisher Scientific (Waltham, MA, USA). Healthy volunteers with no syphilis infection were recruited and were confirmed to be seronegative for syphilis by serological tests conducted by the Second Affiliated Hospital of University of South China.

The NF‐κB inhibitor QNZ was purchased from MCE.

### 2.2 Recombinant protein expression and purification

After removing the signal peptide (1‐18aa), the Tp92 fragment was expressed in *Escherichia coli* and purified with Ni‐NTA columns (Darmstadt, Germany) as described previously.^17^ *E. coli* endotoxins were removed to eliminate potential contamination using polymyxin B‐agarose in accordance with the manufacturer's instructions (Toxin Eraser™ Endotoxin Removal Kit; Toxin Eraser™ Endotoxin Removal Kit; GenScript, NanJin, China (NJ, USA). The final preparations of recombinant Tp92 contained less than 0.25 endotoxin units/μg of protein, corresponding to less than 0.1 EU/mL of the same amount of *E. coli* LPS. The control outer membrane protein Tp0663 was provided by Xu et al.^18^

### 2.3 Cells

Human acute monocytic leukaemia THP‐1 cells were purchased from the American Type Culture Collection (Manassas, VA, USA) and cultured in RPMI 1640 medium, supplemented with 10% foetal bovine serum (Thermo Fisher Scientific) at 37°C and in a 5% CO_2_ atmosphere. Fresh whole blood containing the anticoagulant heparin was mixed with phosphate‐buffered saline (PBS) at a ratio of 1:1 and then added gently into an equal volume of Ficoll‐Paque Premium (STEMCELL Technologies, Vancouver, BC, Canada). After centrifugation at 1000 *g* for 30 minutes, PBMCs were gently washed with PBS twice before discarding the supernatant. Then, the PBMCs were resuspended with RPMI 1640 medium for further use. A hemocytometer was used to count the cells.

RNA interference was performed to silence the TLR2 and TLR4 genes (psiRNA‐hTLR2 and psiRNA‐hTLR4; InvivoGen, San Diego, CA, USA). THP‐1 cells (1 × 10^4^) were plated in 96‐well plates and pretreated with 0.2 μg of psiRNA and Lipofectamine 2000 (Thermo Fisher Scientific) for 24 hours. In addition, THP‐1 cells (1 × 10^5^) were also plated into 6‐well plates and pretreated with 2.0 μg of psiRNA and Lipofectamine 2000 (Thermo Fisher Scientific) for 24 hours.

### 2.4 Acridine orange/ethidium bromide fluorescence assay

THP‐1 cells (5 × 10^4^/well) were plated in 24‐well plates and stimulated with different concentrations of Tp92 for different periods of time. Then, 10 μL of the dyes acridine orange (AO) and ethidium bromide (EB) (1:1 ratio; Thermo Fisher Scientific) was added into each well. After 10 minutes, cell membrane damage was observed by using a fluorescence microscope (magnification, 200×; Nikon, Tokyo, Japan). Green fluorescence indicated that the cell membrane was intact, while red fluorescence indicated that the cell membrane was damaged.

### 2.5 Hoechst33342 staining

THP‐1 cells (5 × 10^4^/well) were plated in 24‐well plates and stimulated with different concentrations of Tp92 for 12 hours. Then, the cells were harvested and washed with PBS twice before being resuspended in 100 μL of PBS. Hoechst33342 dye (5 g/mL; Thermo Fisher Scientific) was added before incubation at 37°C for 15 minutes. After washing with PBS twice, the cells were resuspended in PBS and plated in fresh 24‐well plates for observation of blue fluorescence under a fluorescence microscope (magnification, 400×; Nikon).

### 2.6 Flow cytometry

THP‐1 cells (1 × 10^5^/well) were plated in six‐well plates and stimulated with different concentrations of Tp92 for different periods of time. After harvesting, the cells were subjected to flow cytometry according to the manufacturer's protocol for the fluorescein isothiocyanate (FITC)‐annexin V/propidium iodide kit for the detection of apoptosis (BD Biosciences, Franklin Lakes, NJ, USA). A BD FACSCalibur instrument (BD Biosciences) and FlowJo 7.6 were used to obtain and analyse the data.

THP‐1 cells (1 × 10^5^/well) were plated in 6‐well plates and treated with PBS, Tp92 (5 μg/mL) or carbonyl cyanide 3‐chlorophenylhydrazone for 12 hours. After harvesting, the cells were subjected to flow cytometry according to the protocol for the JC‐1 assay kit (BD Biosciences). The changes in mitochondrial membrane potential were also measured. A BD FACSCalibur instrument (BD Biosciences) and FlowJo 7.6 were used to obtain and analyse the data.

THP‐1 cells (1 × 10^5^/well) were plated in 6‐well plates and treated with PBS, Tp92 (5 μg/mL) or Rosup for 12 hours. After harvesting, the cells were subjected to flow cytometry according to the protocol for the reactive oxygen species (ROS) assay kit (BD Biosciences) to determine ROS levels. A BD FACSCalibur instrument (BD Biosciences) and FlowJo 7.6 were used to obtain and analyse the data. After resuspension, PBMCs were plated into six‐well plates and treated with PBS or Tp92 (5 μg/mL) for 12 hours. After centrifugation at 600 *g*, the cells were washed with PBS twice and resuspended in 100 μL of PBS. Then, the cells were incubated with FITC‐labelled anti‐CD14, anti‐HLA‐DR and anti‐CD3 fluorescence antibodies (BioLegend, SanDiego, CA, USA) at 4°C in the dark for 2 hours. After being washed with PBS twice, the cells were resuspended in 300 μL of PBS before flow cytometry (BD Biosciences). A BD FACSCalibur instrument (BD Biosciences) and FlowJo 7.6 were used to obtain and analyse the data.

### 2.7 Cell counting kit‐8 assay

Total cell death rate was examined using cell counting kit‐8 (CCK‐8) assay. The samples were processed according to the protocol of the CCK‐8 assay kit (Beyotime, Shanghai, China) before determination of absorbance on a reader. After subtracting the absorbance of the blank control, the absorbance values of the samples were used to calculate the total cell death rate.

### 2.8 Lactate dehydrogenase release assay

The samples were processed according to the protocol for the lactate dehydrogenase (LDH) release assay kit (Beyotime) before determination of the absorbance on a reader. After subtracting the absorbance of the blank control, the absorbance values of the samples were used to calculate the LDH release rate.

### 2.9 Measurement of caspase enzyme activity

THP‐1 cells (1 × 10^5^/well) were plated in six‐well plates and treated with Tp92 (5 μg/mL) for 12 hours. The cells were harvested and washed with PBS twice. After the samples were processed according to the manufacturer's instructions for the caspase activity detection kit (Beyotime), the absorbance of each well was measured on a reader. After subtracting the absorbance of the blank control, the absorbance values of the samples were used to calculate the caspase enzyme activity (U).

### 2.10 Western blotting

Cells were centrifuged and collected before lysis with radio‐immunoprecipitation assay lysis buffer (Thermo Fisher Scientific). The supernatant was used to determine protein concentration by the BCA protein concentration determination kit (Thermo Fisher Scientific). Then, loading buffer was added to the samples before boiling at 100°C for 5 minutes. Then, 10% or 12% sodium dodecyl sulfate‐polyacrylamide gel electrophoresis was performed to separate proteins with different molecular weights. The resolved proteins were transferred to polyvinylidene difluoride membranes (Millipore, Billerica, MA, USA) on ice (100 V, 1 hour) and blocked with 5% skim milk at room temperature for 1 hour. Then, the membranes were incubated with rabbit anti‐caspase‐1, anti‐pro‐caspase‐1, anti‐GSDMD, anti‐GAPDH, and anti‐TLR2 monoclonal primary antibodies (1:1000; Abcam, Cambridge, MA, USA); rabbit anti‐TLR4 polyclonal primary antibodies (1:500; Abcam); and rabbit anti‐NF‐κB p‐p65 polyclonal antibodies (1:1000; Abcam) at 4°C overnight. After extensive washing, the membranes were incubated with polyclonal goat anti‐rabbit horseradish peroxidase‐conjugated secondary antibody (1:5000; Proteintech, Chicago, IL, USA) for 2 hours at 37°C. After washing, enhanced chemiluminescence was performed according to the manufacturer's protocol for the Pierce ECL Plus reagent (Thermo Fisher Scientific). The relative expression of the target proteins was calculated against the grayscale of GAPDH.

### 2.11 Enzyme‐linked immunosorbent assay

To determine the secretion levels of tumour necrosis factor (TNF)‐α, interleukin (IL)‐1β, IL‐6, IL‐8,IL‐10, IL‐18 and MCP‐1, the supernatants of the treated cells were collected and subjected to an enzyme‐linked immunosorbent assay (ELISA) according to the manufacturer's instructions (Neobioscience, Shenzhen, China).

### 2.12 *Treponema pallidum* propagation

The propagation of *T. pallidum* was performed in accordance with the method described by Lukehart and Marra.^19^ Subsequently, *T. pallidum w*as extracted from testes under the appropriate conditions.

### 2.13 Statistical analysis

The results were analysed using SPSS 18.0 statistical software (IBM, Armonk, NY, USA). All data were expressed as means ± SDs. Student's *t* test was used for comparisons between two groups. A nonparametric statistical test was used for comparisons across three conditions. Differences with *P*‐values less than 0.05 were considered statistically significant. All tests were performed in triplicate.

## 3 RESULTS

### 3.1 Stimulation by the Tp92 protein induces THP‐1 cell death

**Figure 1:**
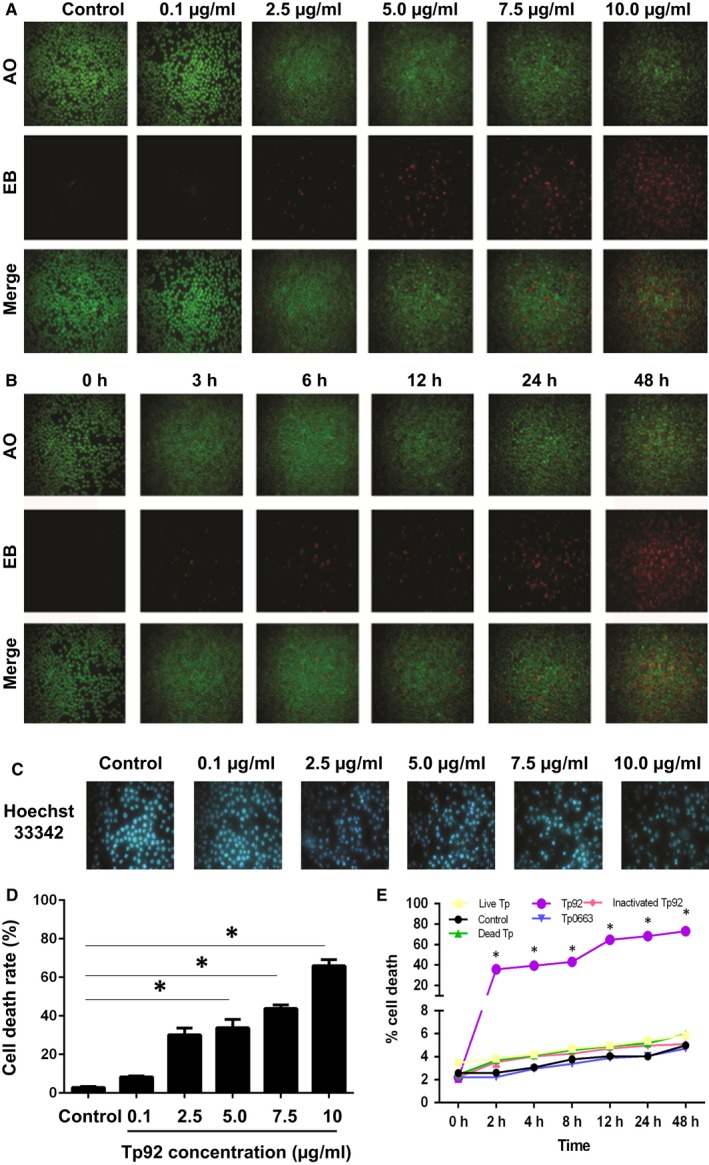
Effect of the Tp92 protein on THP‐1 cell death. A and B, Effect of different (A) concentrations or (B) durations of Tp92 treatment on the integrity of THP‐1 cell membranes. The cells were treated with (A) 0, 0.1, 2.5, 5.0, 7.5 or 10.0 μg/mL Tp92 for 12 hours or (B) 5.0 μg/mL Tp92 for 0, 3, 6, 12, 24 or 48 hours. AO/EB staining was performed, and the cells were observed under a fluorescence microscope. Red indicates cells with damaged membrane, while green indicates intact cells. C, Effect of different concentrations of the Tp92 protein on the nuclei of THP‐1 cells. The cells were stimulated with 0, 0.1, 2.5, 5.0, 7.5 or 10.0 μg/mL Tp92 for 12 hours, and the nuclei were stained with Hoechst33342 dye and observed under a fluorescence microscope. D and E, Effect of different (D) concentrations or (E) durations of Tp92 treatment on the total death rates of THP‐1 cells. The cells were treated with (D) 0, 0.1, 2.5, 5.0, 7.5 or 10.0 μg/mL Tp92 for 12 hours or (E) 1 × 10^3^ live Tp (*Treponema pallidum*), 1 × 10^3^ dead Tp, 5.0 μg/mL Tp0663 (*T. pallidum outer membrane protein*), 5.0 μg/mL Tp92 and 5.0 μg/mL inactivated Tp92 (heat inactivation) for 0, 2, 4, 8, 12, 24 or 48 hours. The CCK‐8 assay was used to determine the total death rates of the THP‐1 cells. Data are expressed as the means ± standard deviations of three replicates. *P < 0.05 compared with the control

To test whether Tp92 induces THP‐1 cell death, the cells were subjected to AO, EB or Hoechst33342 staining. The results of AO/EB staining showed that cell membrane damage was dependent on the concentration and duration of Tp92 treatment, and treatment with a concentration of 10 μg/mL (Figure 1A) or for a duration of 48 hours (Figure 1B) resulted in the maximum number of damaged cells. Upon increasing the Tp92 concentration, the nucleus also appeared to be broken and slightly shrunken (Figure 1C). In addition, the total cell death rate was also dependent on the concentration and duration of Tp92 treatment, and a concentration of 10 μg/mL (Figure 1D) or a duration of 48 hours (Figure 1E) led to the highest total death rate of THP‐1 cells. In contrast, the total cell death rate in the live and dead Tp groups, inactivated Tp92 (heat inactivation) group and Tp0663 group did not increase. These results suggested that stimulation by the Tp92 protein induces THP‐1 cell death.

### 3.2 The Tp92 protein may induce atypical pyroptosis of THP‐1 cells via the pro‐caspase‐1 pathway

**Figure 2:**
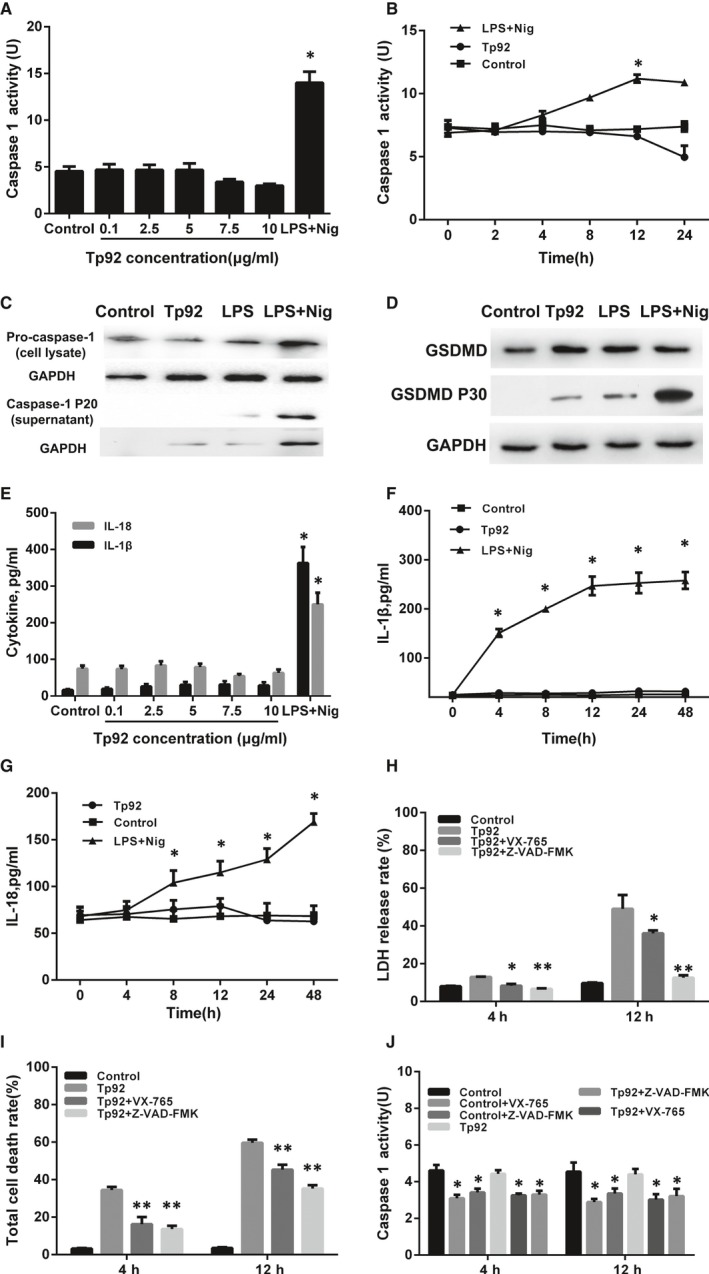
Effect of the Tp92 protein on the pyroptosis of THP‐1 cells. A and B, Effect of different (A) concentrations and (B) durations of Tp92 or LPS+Nig treatment on caspase‐1 activity. THP‐1 cells were treated with (A) 0, 0.1, 2.5, 5.0, 7.5 or 10.0 μg/mL Tp92 or LPS (1 μg/mL)+Nig (5 μM) for 12 hours or (B) LPS (1 μg/mL)+Nig (5 μM) or 5.0 μg/mL Tp92 for 0, 2, 4, 8, 12 or 24 hours. *P < 0.05 compared with the control. C and D, Effect of Tp92, LPS or LPS+Nig on (C) caspase‐1, pro‐caspase‐1 and (D) GSDMD protein expression determined by Western blotting. THP‐1 cells were treated with Tp92 (5 μg/mL), LPS (1 μg/mL) or LPS (1 μg/mL)+Nig (5 μM) for 12 hours before Western blotting. E‐G, Effect of different (E) concentrations and (F and G) durations of Tp92 or LPS+Nig treatments on the concentrations of IL‐1β and IL‐18 measured by ELISA. THP‐1 cells were treated with (E) 0, 0.1, 2.5, 5.0, 7.5 or 10.0 μg/mL Tp92 or LPS (1 μg/mL)+Nig (5 μM) for 12 hours or (F and G) LPS (1 μg/mL)+Nig (5 μM) or 5.0 μg/mL Tp92 for 0, 4, 8, 12, 24 or 48 hours. *P < 0.05 compared with the control. H‐J, Effect of the caspase‐1‐specific inhibitor VX‐765 or caspase inhibitor Z‐VAD‐FMK on the (H) LDH release rate, (I) total cell death rate and (J) caspase‐1 activity. THP‐1 cells were pretreated with VX‐765 (50 μM) or Z‐VAD‐FMK (100 μM) for 1 hour before being treated with Tp92 (5 μg/mL) for 12 hours. At 4 and 12 hours, the LDH release rate, total cell death rate and casepase‐1 activity were measured and expressed as the means ± standard deviations of three replicates. *P < 0.05 and **P < 0.01 compared with the Tp92 group

To examine whether Tp92 induces pyroptosis, caspase‐1 enzyme activity was tested. The data showed that different concentrations or durations of Tp92 treatment failed to alter the caspase‐1 enzyme activity (no significant differences were observed); however, the caspase‐1 enzyme activity in the LPS+Nig group was significantly higher than that in the control group (*P* < 0.05) (Figure 2A and B). Activated caspase‐1 can be released from cells through the secretory pathway. Western blotting showed that the caspase‐1 protein level in the supernatant in the Tp92 group was not different from that in the control group, but the levels in the LPS and LPS+Nig groups were higher than the level in the control group. However, the pro‐caspase‐1 protein levels in the Tp92, LPS and LPS+Nig groups were not significantly different from the level in the control group (Figure 2C). Notably, GSDMD P30 levels in the Tp92, LPS and LPS+Nig groups were higher than the level in the control group, suggesting that GSDMD was cleaved in the three groups (Figure 2D). The ELISA showed that different concentrations or durations of Tp92 treatment did not alter the concentration of IL‐1β or IL‐18 (no significant differences were observed); however, the IL‐1β and IL‐18 levels in the LPS+Nig group were significantly higher than those in the control group (*P* < 0.05) (Figure 2E‐G). Furthermore, the cells were treated with the caspase‐1‐specific inhibitor VX‐765 or pan‐caspase inhibitor Z‐VAD‐FMK before examining LDH release, total death rate and caspase‐1 activity. After treatment with Tp92 for 4 or 12 hours, the LDH release rates in the Tp92+VX‐765 and Tp92+Z‐VAD‐FMK groups were significantly lower than the rate in the Tp92 group (*P* < 0.05) (Figure 2H). In addition, the total cell death rates in the Tp92+VX‐765 and Tp92+Z‐VAD‐FMK groups were significantly lower than the rate in the Tp92 group (*P* < 0.05) (Figure 2I). Similarly, caspase‐1 activity in the Tp92+VX‐765 and Tp92+Z‐VAD‐FMK groups was significantly lower than that in the Tp92 group (*P* < 0.05) (Figure 2J). These results indicated that the Tp92 protein may induces atypical pyroptosis of THP‐1 cells via the pro‐caspase‐1 pathway.

### 3.3 The Tp92 protein causes apoptosis of THP‐1 cells via the RIPK1/caspase‐8/caspase‐3 pathway

**Figure 3:**
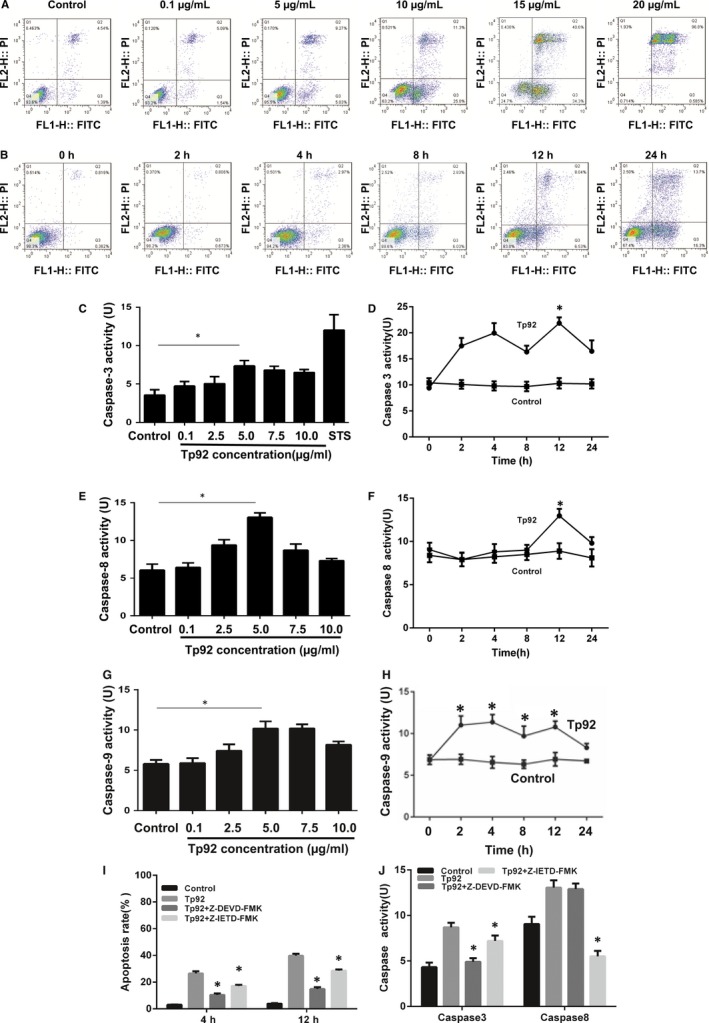
Effect of the Tp92 protein on the apoptosis of THP‐1 cells. A and B, Effect of different (A) concentrations and (B) durations of Tp92 treatment on the apoptosis of THP‐1 cells, as determined by flow cytometry. The cells were treated with (A) 0, 0.1, 5.0, 10.0, 15.0 or 20.0 μg/mL Tp92 for 12 hours or (B) 5.0 μg/mL Tp92 for 0, 2, 4, 8, 12 or 24 hours. C‐H, Effect of different (C, E, G) concentrations and (D, F, H) durations of Tp92 treatment on the activities of caspase‐3, ‐8 and ‐9. STS (0.1 μM) was also used to treat THP‐1 cells to study the activity of caspase‐3. *P < 0.05 compared with the control. I, Effect of caspase‐3 and caspase‐8 inhibitors on the apoptosis of THP‐1 cells. Cells were pretreated with the caspase‐3 inhibitor Z‐DEVD‐FMK (10 μM) or caspase‐8 inhibitor Z‐IETD‐FMK (1 μL/mL) for 1 hour and then treated with Tp92 (5 μg/mL) for 4 or 12 hours before measuring the apoptotic rate. *P < 0.05 compared with the Tp92 group. J, Effect of caspase‐3 and caspase‐8 inhibitors on the activities of caspase‐3 and caspase‐8. Cells were pretreated with Z‐DEVD‐FMK (10 μM) or Z‐IETD‐FMK (1 μL/mL) for 1 hour and then treated with Tp92 (5 μg/mL) for 12 hours before testing the activities of caspase‐3 and caspase‐8. *P < 0.05 compared with the Tp92 group

**Figure 4:**
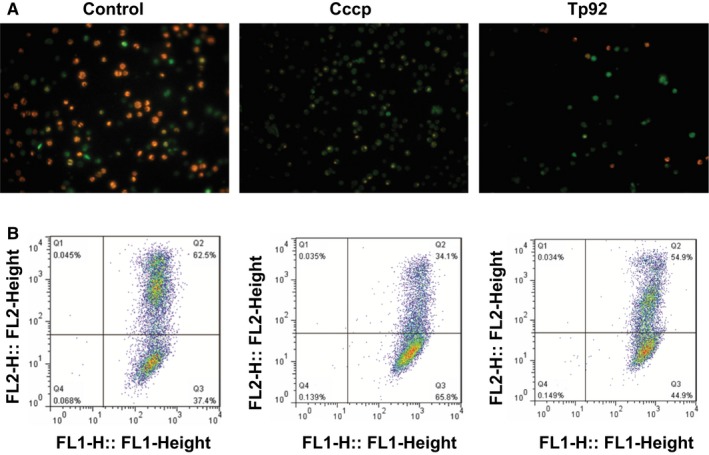
Effect of Tp92 on mitochondria. A, Fluorescence microscopy of mitochondria in THP‐1 cells (magnification, 200 × ). B, Mitochondrial transmembrane potential determined by flow cytometry. THP‐1 cells were treated with carbonyl cyanide 3‐chlorophenylhydrazone (Cccp) or Tp92 (5.0 μg/mL) for 12 hours

**Figure 5:**
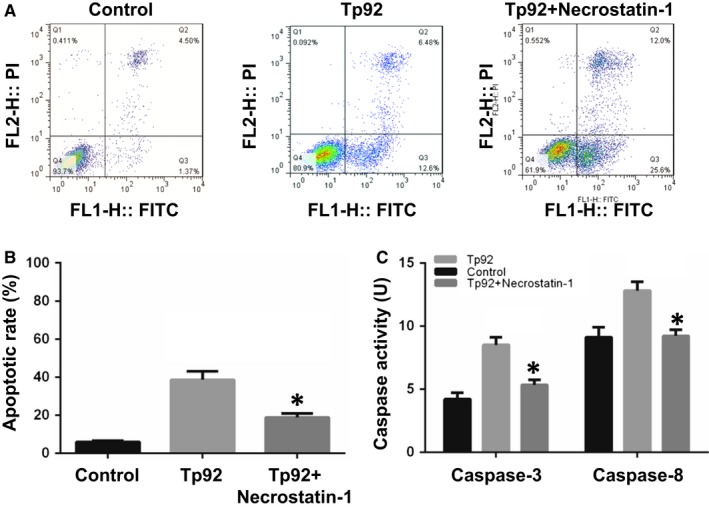
Effect of the RIPK1 inhibitor necrostatin‐1 on (A and B) apoptosis and (C) caspase activity. A and B, Apoptotic rate of THP‐1 cells determined by flow cytometry. B, Activities of caspase‐3 and caspase‐8. The cells were pretreated with necrostatin‐1 (10 μM) and then incubated with Tp92 (5 μg/mL) for 12 hours. *P < 0.05 compared with the Tp92 group

**Figure 6:**
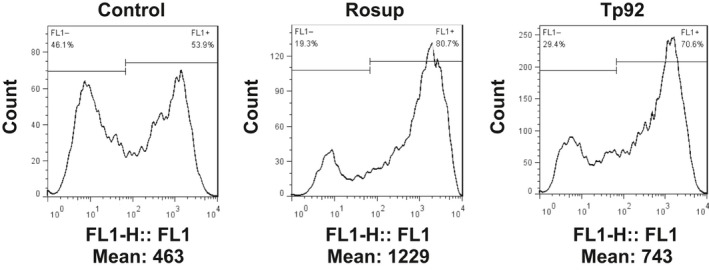
Effect of Tp92 on the ROS levels in THP‐1 cells. The cells were treated with Rosup (1 μg/mL) or Tp92 (5 μg/mL) for 12 hours. Average fluorescence intensity was measured by flow cytometry

To study how Tp92 affects the apoptosis of THP‐1 cells, flow cytometry was performed. The data showed that the Tp92 protein induced the apoptosis of THP‐1 cells in a concentration‐ and time‐dependent manner (Figure 3A and B). In addition, the activities of caspase‐3 and caspase‐8 in the presence of high concentrations of the Tp92 protein were significantly higher than those in the control (*P* < 0.05) (Figure 3C and E), and treatment with Tp92 (5.0 μg/mL) for 12 hours resulted in peaks corresponding to caspase‐3 and caspase‐8 activities (*P* < 0.05) (Figure 3D and F). Similarly, caspase‐9 activity was also increased upon treatment with the Tp92 protein (Figure 3G and H). Furthermore, pretreatment with the caspase‐3 inhibitor Z‐DEVD‐FMK or caspase‐8 inhibitor Z‐IETD‐FMK reduced the apoptotic rates of the THP‐1 cells (*P* < 0.05) (Figure 3I). Interestingly, treatment with the caspase‐8 inhibitor Z‐IETD‐FMK reduced the activities of both caspase‐3 and caspase‐8 (*P* < 0.05). In contrast, the caspase‐3 inhibitor Z‐DEVD‐FMK only reduced caspase‐3 activity (*P* < 0.05) (Figure 3J). Moreover, treatment with Tp92 reduced the mitochondrial transmembrane potential, suggesting that Tp92 might also activate the mitochondrial apoptotic pathway in THP‐1 cells (Figure 4). To test whether RIPK1 is involved the apoptosis of THP‐1 cells induced by Tp92, THP‐1 cells were pretreated with the RIPK1 inhibitor necrostatin‐1 before determining the apoptotic rate. The data showed that the apoptotic rate in the Tp92+necrostatin‐1 group was significantly lower than that in the Tp92 group (*P* < 0.05) (Figure 5A and B). In addition, the activities of caspase‐3 and caspase‐8 in the Tp92+necrostatin‐1 group were significantly lower than those in the Tp92 group (*P* < 0.05) (Figure 5C). The flow cytometry data also revealed that the ROS level in the Tp92 group was higher than that in the control group (*P* < 0.05) (Figure 6). These results suggested that the Tp92 protein causes apoptosis of THP‐1 cells via the RIPK1/caspase‐8/caspase‐3 pathway.

### 3.4 Tp92 mediates THP‐1 cell death by recognizing CD14 and/or TLR2 on the cell surface

**Figure 7:**
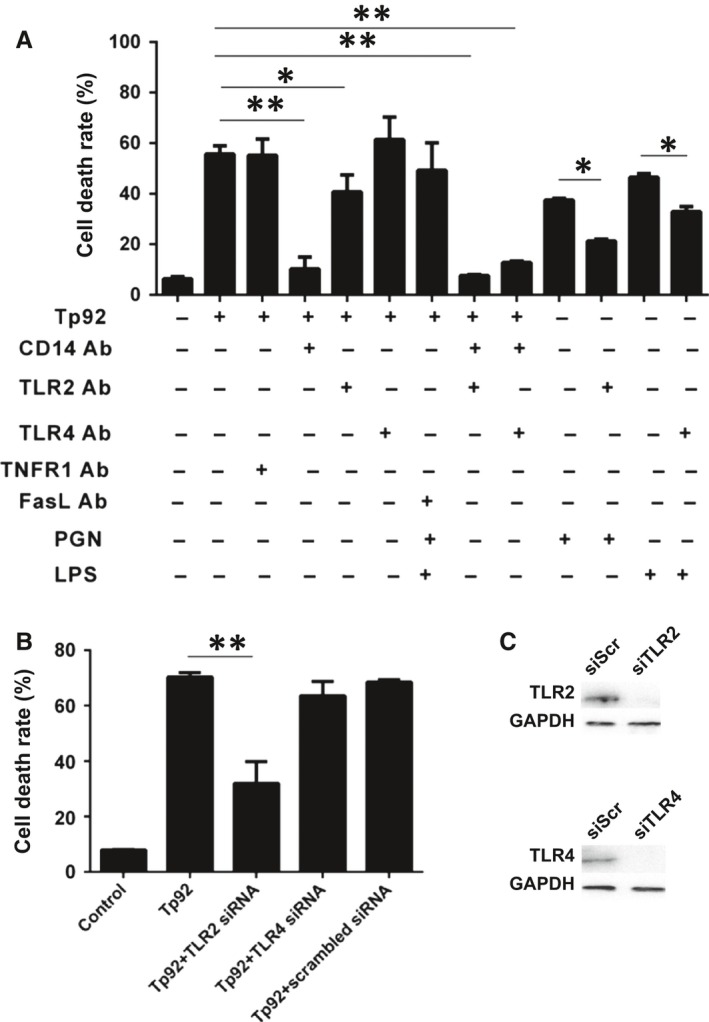
Tp92 mediates THP‐1 cell death by recognizing CD14 and/or TLR2 on cell surfaces. A, Death rates of THP‐1 cells pretreated with TNFR1, FasL, TLR4, CD14 or TLR2‐neutralizing antibodies (10 μg/mL each) for 1 hour. The cells were treated with Tp92 (5.0 μg/mL), LPS (1 μg/mL) or PGN (10 μg/mL) for 12 hours, and the CCK‐8 assay was performed to determine the total cell death rate. B, Death rates of THP‐1 cells pretreated with TLR2 (2 μg/mL) or TLR4 (2 μg/mL) interference plasmids for 24 hours. The cells were treated with Tp92 (5 μg/mL) for 12 hours, and the CCK‐8 assay was carried out to measure the total cell death rate. Data are expressed as the means ± standard deviations (n = 3). *P < 0.05 and **P < 0.01. C, Expression of TLR2 and TLR4 proteins in THP‐1 cells pretreated with TLR2 and TLR4 interference plasmids for 24 hours. Western blotting was used to determine protein expression

To investigate the mechanism via which the Tp92 protein triggers the death of THP‐1 cells, the cells were treated with TNFR1‐, FasL‐, TLR4‐, CD14‐ and TLR2‐neutralizing antibodies. The data showed that the cell death rate in the Tp92+anti‐CD14 Ab group was significantly lower than that in the Tp92 group (*P* < 0.05) but was not different from that in the negative control (*P* > 0.05). Similarly, the Tp92+anti‐TLR2 Ab, Tp92+anti‐TLR2 Ab+anti‐CD14 Ab, Tp92+anti‐TLR4Ab+anti‐CD14 groups also exhibited significantly reduced cell death rates compared with the cell date rate in the Tp92 group (*P* < 0.05). However, the cell death rates in the other groups treated with neutralizing antibodies were not significantly different from the rate in the Tp92 group (Figure 7A). Pretreatment with the TLR2 dominant negative plasmid or TLR4 interference plasmid showed that the cell death rate in the Tp92+TLR2 siRNA group was significantly lower than that in the Tp92 group (*P* < 0.01); however, the cell death rate in the Tp92+TLR4 siRNA group was not significantly different from that in the Tp92 group (*P* > 0.05) (Figure 7B). Western blotting showed that the siRNA reduced the expression of TLR2 and TLR4 (Figure 7C). These results indicate that Tp92 mediates THP‐1 cell death by recognizing CD14 and/or TLR2 on the cell surface.

### 3.5 Tp92 reduces the number of monocytes and induces the secretion of IL‐8 from PBMCs

**Figure 8:**
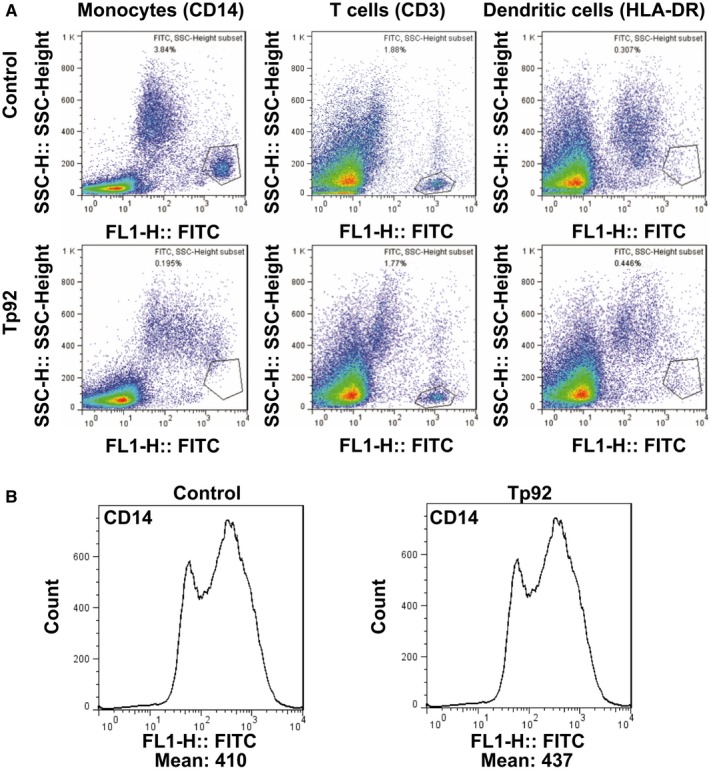
Effect of the Tp92 protein on PBMCs. A, Fluorescence intensity of monocytes (CD14), T cells (CD3) and dendritic cells (HLA‐DR) among PBMCs. The cells were treated with Tp92 (5 μg/mL) for 12 hours and then incubated with FITC‐conjugated anti‐CD14, anti‐CD3 or anti‐HLA‐DR antibody at 4°C in the dark for 1 hour before flow cytometry. B, CD14 expression on the surfaces of THP‐1 cells pretreated with Tp92. The cells were treated with Tp92 (5 μg/mL) for 12 hours and then incubated with FITC‐conjugated anti‐CD14 antibody at 4°C in the dark for 1 hour before flow cytometry. *P < 0.05

**Figure 9:**
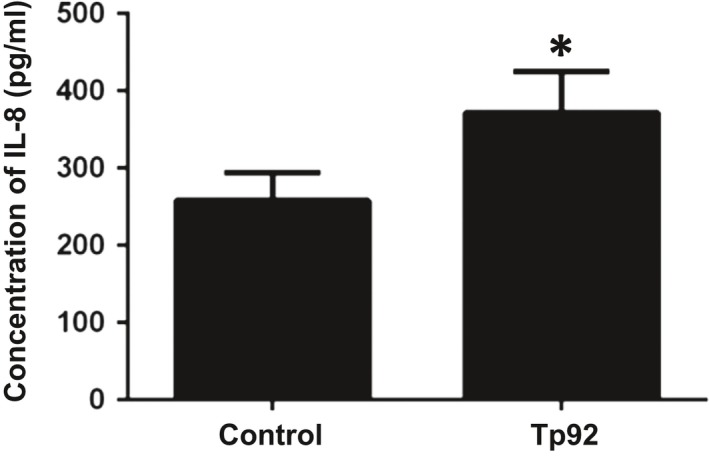
Effect of the Tp92 protein on PBMCs. Concentration of IL‐8 secreted by PBMCs, as determined by ELISA. The cells were treated with Tp92 (5 μg/mL) for 12 hours before ELISA. *P < 0.05

To study whether the Tp92 protein affects PBMCs in the body, PBMCs were coincubated with anti‐CD14, anti‐HLA‐DR, or anti‐CD3 fluorescent antibodies. The data showed that the fluorescence intensities of HLA‐DR and CD3 in PBMCs treated with Tp92 was not significantly different from those in untreated PBMCs (no significant differences were observed); however, the CD14 fluorescence intensity in PBMCs treated with Tp92 was significantly lower than that in untreated PBMCs (*P* < 0.05) (Figure 8A). Furthermore, coincubation of the anti‐CD14 fluorescence antibody with Tp92‐pretreated THP‐1 cells showed that CD14 expression on the surfaces of THP‐1 cells pretreated with Tp92 was not significantly different from that on the surfaces of untreated THP‐1 cells (no significant differences were observed) (Figure 8B). The results showed that Tp92 treatment did not lead to a decrease in levels in single monocytes. As shown in Figure 8A, the decrease in CD14 levels could only be caused by a decrease in the total number of monocytes. The amount of IL‐8 secreted by the PBMCs was determined by ELISA. The results showed that Tp92 induced IL‐8 secretion from PBMCs (Figure 9).

### 3.6 Tp92 induces the cellular secretion of IL‐8 via the NF‐κB pathway and CD14 and/or TLR2 on the surfaces of THP‐1 cells

**Figure 10:**
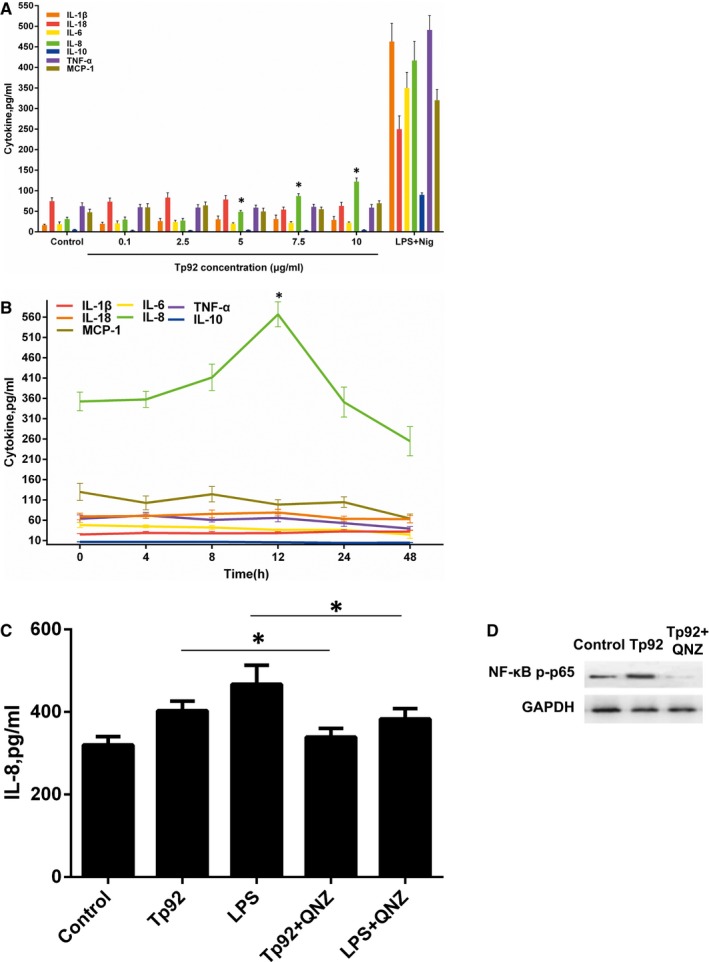
Effect of Tp92 on the secretion of cytokines by THP‐1 cells. A and B, Concentrations of secreted cytokines determined by ELISA. The cells were treated with (A) 0, 0.1, 2.5, 5.0, 7.5 or 10.0 μg/mL Tp92 or LPS (1 μg/mL)+Nig(5 μM) for 12 hours or (B) 5.0 μg/mL Tp92 for 0, 4, 8, 12, 24 or 48 hours. *P < 005 compared with (A) PBS or (B) 0 hour. C and D, Effect of the NF‐κB inhibitor QNZ on the secretion of IL‐8. C, Concentration of secreted IL‐8 measured by ELISA. The cells were pretreated with QNZ (10 nM) for 1 hour before treatment with the control (DMSO), Tp92 (5 μg/mL) or LPS (1 μg/mL) for 12 hours. *P < 005. D, NF‐κB protein expression in cells treated with Tp92 (5 μg/mL) or Tp92 (5 μg/mL)+QNZ (10 nM)

The effect on the secretion of cytokines was measured by an ELISA kit after THP‐1 cells were treated with Tp92. The results showed that Tp92 failed to increase the TNF‐α, IL‐1β, IL‐6, IL‐10, IL‐18 and MCP‐1 levels and slightly elevated the IL‐8 levels in THP‐1 cells (Figure 10A and B). The amount of IL‐8 secreted was tested by ELISA, and NF‐κB protein expression was determined by Western blotting after the cells were pretreated with the NF‐κB inhibitor QNZ. The results showed that Tp92 up‐regulated IL‐8 and NF‐κB protein levels, and the NF‐κB inhibitor down‐regulated the IL‐8 and NF‐κB protein levels (Figure 10C and D).

**Figure 11:**
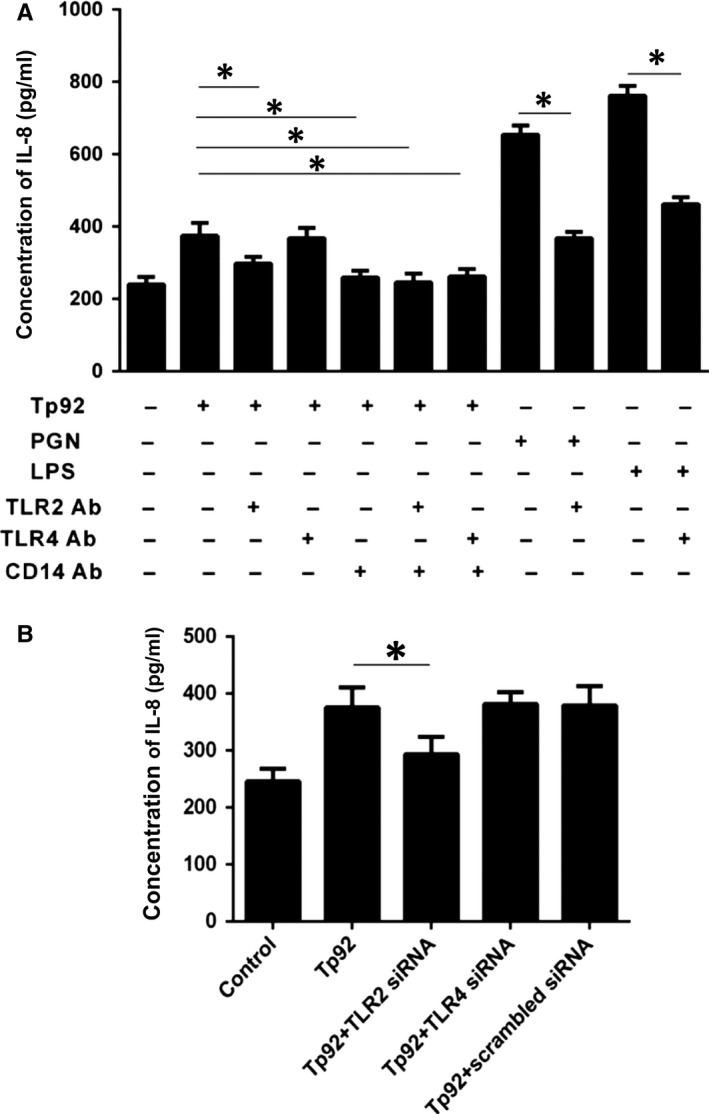
Mechanism by which the Tp92 protein mediates IL‐8 secretion. A and B, Concentration of secreted IL‐8 measured by ELISA. A, The cells were pretreated with CD14 (10 μg/mL), TLR2 (10 μg/mL) or TLR4 (10 μg/mL) for 1 hour before treatment with Tp92 (5 μg/mL), PGN (10 μg/mL), or LPS (1 μg/mL) for 12 hours. *P < 005. B, The cells were pretreated with the siRNA of TLR2 (2 μg/mL) and TLR4 (2 μg/mL) for 24 hours before treatment with Tp92 (5 μg/mL) for 12 hours. *P < 005

The amount of IL‐8 secreted was measured by ELISA after the cells were treated with neutralizing antibodies or pretreated with CD14, TLR2 or TLR4 interference plasmids. The results showed that only the CD14‐ and TLR2‐neutralizing antibodies and the TLR2 interference plasmid could effectively reduce the secretion of IL‐8 (Figure 11A and B).

## 4 DISCUSSION

CD14 is a glycosyl‐phosphatidyl inositol‐anchored protein located on the surfaces of monocytes and macrophages, and this protein identifies and binds to specific molecular structures that are commonly found on the surfaces of pathogenic microorganisms, such as LPS on Gram‐negative bacteria; pathogen‐associated molecular patterns (PAMPs) such as PGN and lipoteichoic acid (LTA) on gram‐positive bacteria, or lipoproteins on *Borrelia burgdorferi* and *T. pallidum*.^20^, ^21^, ^22^ CD14 exerts its effect on cell surfaces together with TLRs, which can also recognize PAMPs. CD14 can recognize LPS together with MD2/TLR4^23^ and can recognize PGN, LTA or lipoprotein together with TLR2.^24^, ^25^, ^26^, ^27^, ^28^ In addition, CD14 is a common receptor of TLR7 and TLR9.^29^ When the LPS concentration is low, CD14 helps TLR4 identify LPS. When the LPS concentration is high, CD14 recognizes LPS by itself, as observed for the recognition of PGN by TLR2.^30^ CD14 can also recognize the outer membrane proteins of some specific pathogens. For example, the *Borrelia burgdorferi* outer membrane protein OspA competes with LPS to bind to CD14, suggesting that the binding site of OspA is similar to that of LPS.^31^ Although LPS has not been found in *T. pallidum*, the outer membrane protein Tp92 may play a role similar to that of LPS.

The present study shows that the outer membrane protein Tp92 of *T. pallidum* mediates cell death by recognizing CD14 and/or TLR2 on the surfaces of human mononuclear cells, and the cell death rate was enhanced when the concentration or duration of Tp92 treatment was increased.

In the present study, we co‐cultured with THP‐1 using live or dead *T. pallidum*, Tp0663, Tp92 or inactivated Tp92 (heat inactivation) respectively. The results show that only Tp92 could cause cell death. Tp0663 and Tp92 belong to a group of surface‐exposed, rare *Treponema* outer membrane proteins and are the part of the innate immune system of *T. pallidum* that first encounters the host environment.^32^ However, not all of the outer membrane proteins of *T. pallidum* can cause the death of monocytes. In the present experiment, only the recombinant outer membrane protein Tp92 could cause cell death, while the control outer membrane protein Tp0663 did not show similar results, indicating that Tp92 may play an important role in pathogenic infection. Interestingly, although the recombinant protein Tp92 could cause monocyte (THP‐1) death, live and dead *T. pallidum* did not cause cell death. There may be several explanations for this finding. One of the main explanations is that Tp92 is a rare outer membrane protein on the surfaces of *T. pallidum* cells.^14^ In addition, it is possible that natural Tp92 on *T. pallidum* cell surfaces could not induce THP‐1 cell death because the functional areas of the protein required for the induction of cell death were directly exposed because of the spatial conformation of the protein. Furthermore, it is possible that the natural Tp92 protein on *T. pallidum* cell surfaces, similar to bacterial endotoxins, cannot be released under normal bacterial living conditions and can cause cell death only by degrading and exposing the death domain when the *T. pallidum* bacterial cells are lysed because of various factors. On the other hand, *T. pallidum* cannot be cultured in vitro, and based on the specificity of the pathogen, the survival rate of *T. pallidum* isolated from the New Zealand rabbit upon interaction with THP‐1 could not be determined. Previous observations suggested that *T. pallidum* was unable to survive in vitro for long durations, and the exact survival time of *T. pallidum* was not indicated in the related literature.^33^ Therefore, the cellular experiments involving interactions between *T. pallidum* and THP‐1 in vitro may not truly reflect the *T. pallidum* infection process in the body. Currently, we are unable to explore the function of Tp92 by gene knockout in live *T. pallidum*, and the explanation behind this property requires further exploration.

During pathogenic infection, the main mechanisms of host cell death are pyroptosis and apoptosis.^6^, ^7^ Pyroptosis is a type of inflammation‐related cell death that includes typical pyroptosis and atypical pyroptosis. In typical pyroptosis, activated NLRP3 binds with ASC to accumulate pro‐caspase‐1, which is then cleaved into active caspase‐1. Activated caspase‐1 cleaves the precursors of IL‐1β and IL‐18 to facilitate the maturation and secretion of these proteins. On the other hand, activated caspase‐1 cleaves GSDMD, and the resultant GSDMD‐N is then transferred to the cell membrane, where nonselective pores are formed and the cell membrane is damaged, finally leading to cell death.^34^, ^35^ In atypical pyroptosis, the inflammasome NLRC4 directly activates pro‐caspase‐1 without cleaving the precursors of IL‐1β and IL‐18. Then, GSDMD is directly cleaved, and GSDMD‐N is transferred to the cell membrane, leading cell membrane damage and cell death.^36^, ^37^ In the present study, Tp92 treatment failed to elevate the caspase‐1 levels and enzyme activity. Moreover, the secretion of IL‐1β and IL‐18 was not elevated. However, GSDMD was cleaved. After pretreatment with the caspase‐1 inhibitor VX‐765 and caspase inhibitor Z‐VAD‐FMK, the cell death rate was reduced, suggesting that the caspase‐1 inhibitor inhibits cell death. Meanwhile, the THP‐1 membrane was disrupted and LDH release was elevated when Tp92 induced THP‐1 cell death, suggesting that Tp92 possibly causes atypical pyroptosis via pro‐caspase‐1. However, it remains unclear whether Tp92 induces atypical pyroptosis by activating NLRC4.

Apoptosis is a type of cell death that is not related with inflammation but is dependent on caspase. It is believed that caspase‐1, ‐4, ‐5 and ‐11 are associated with pyroptosis, while caspase‐3, ‐6, ‐7, ‐8 and ‐9 are associated with apoptosis.^38^, ^39^, ^40^, ^41^, ^42^ The present study also reveals that Tp92 induces the apoptosis of THP‐1 cells, which is dependent on the concentration and duration of Tp92 treatment. RIPK1 is composed of an N‐terminal kinase domain, an intermediate domain and a C‐terminal death domain. The C‐terminal death domain of RIPK1 can interact with the TNFR1‐associated death domain and Fas‐associated death domain and induces apoptosis by activating caspase‐3, ‐6 and ‐7 via the activation of caspase‐8.^43^, ^44^ In the present study, ROS levels were seen to be elevated during the induction of THP‐1 apoptosis by Tp92. It has been reported that RIPK1 induces the production of ROS,^45^ and ROS are associated with apoptotic signal transduction.^46^ Our data showed that the RIPK1 inhibitor necrostatin‐1 blocks the apoptosis that is induced by Tp92 and inhibits the activities of caspase‐3 and caspase‐8. These results suggested that Tp92 induces THP‐1 cell apoptosis by activating the RIPK1/caspase‐8/caspase‐3 pathway. The results of the present study also show that caspase‐9 is activated and the mitochondrial membrane potential is decreased, suggesting that the mitochondrial apoptosis pathway may also be involved.

RIPK1 is a key molecule in programmed cell death.^47^ When caspase‐8 activation is low or the expression of RIPK3 and mixed lineage kinase domain‐like protein (MLKL) is high, RIPK1 and RIPK3 can activate each other by via cross‐phosphorylation. Activated RIPK3 phosphorylates MLKL, which then acts on ion channels in the cell membrane to induce cell death.^48^, ^49^ In the present study, caspase‐8 activation was elevated by Tp92 treatment, but it is not clear whether Tp92 activated the necroptotic pathway. In future research, we will continue to explore the relationship between Tp92 and necroptosis.

Activation of TNFR, Fas and TNF‐related apoptosis‐inducing ligand receptors (TRAIL‐Rs or DR4/5) stimulates various downstream cell death pathways.^50^ However, only TNFR1 and Fas can induce downstream cell death pathways via RIPK1.^43^, ^51^ In recent years, researchers have found that TLRs can also activate downstream cell death pathways. For example, *Yersinia pestis* binds with TLR4 and triggers the downstream TRIF‐RIPK1‐caspase‐8/FADD‐caspase‐3 pathway to induce apoptosis of host cells.^52^ It is reported that TLR3 and TLR4 can induce apoptosis by regulating the TRIF pathway.^53^ Some studies have shown that some membrane lipoproteins of *T. pallidum* can mediate inflammatory reactions by CD14 and TLR2, but the ability to induce host cell death has not been reported.^54^ Based on the above analysis, TNFR1, FasL, TLR4, CD14 and TLR2 were selected as proteins of interest. Our data demonstrate that THP‐1 death induced by Tp92 is not associated with TNFR1, Fas or TLR4 but is associated with CD14 and/or TLR2. In addition, anti‐CD14 Ab treatment completely inhibits cell death, and anti‐TLR2 Ab treatment partially inhibits cell death. CD14 not only transfers the identified lipoprotein signal to TLR2 but also transfers the identified LPS signal to TLR4.^55^ Our data showed that silencing of TLR2 inhibits cell death, but silencing of TLR4 fails to inhibit cell death, suggesting that the Tp92 protein induces THP‐1 cell death via CD14 and/or TLR2. A study showed that TLR2 activates the inflammasome NLRP3,^56^ but there has been no report on whether TLR2 activates NLRC4 and mediates atypical pyroptosis.

It is believed that there are two ways by which CD14 and/or TLR2 mediates apoptosis. First, TLR2 activates the downstream MyD88, which binds with TRADD and activates the downstream caspase‐8, leading to the activation of caspase‐3 and apoptosis.^57^ Second, TLR2 activates MyD88, which then activates NF‐κB and promotes the production of TNF‐α, triggering the downstream apoptosis pathway via interaction between TNF‐α and TNFR1.^58^ Our data showed that TNF‐α secretion was not elevated, and TNFR1‐ and Fas‐neutralizing antibodies did not block Tp92‐induced cell death, suggesting that THP‐1 cell death induced by Tp92 is not associated with TNFR1. It has been reported that CD14 binds LPS, transfers the signal to MD2 and TLR4, and activates the downstream MyD88 pathway or the TRIF pathway.^59^ Yang et al discovered that stimulation with LPS enhances the TRIF, RIPK1, TRAF3, NF‐κB, IRF7, TNF‐α, IL‐1β and IL‐6 levels in pulmonary alveolar macrophages after CD14 binds with LPS.^60^ The mechanism by which Tp92 induces THP‐1 apoptosis may be that Tp92 recognizes CD14 and CD14 transfers the signal to TLR2, which then activates the RIPK1/caspase‐8/caspase‐3 pathway. It is also possible that CD14 directly activates the TRIF pathway after recognizing Tp92, finally leading to apoptosis.

Pathogens in the early stages of infection not only induce innate immune cell death but can also induce the release of inflammatory factors by the innate immune system. *T. pallidum* mediates the inflammatory reaction and induces the production of proinflammatory cytokines. For example, Tp0751 promotes the secretion of TNF‐α, IL‐1β and IL‐6 by THP‐1 cells via CD14 and TLR2.^61^ A previous study also found that Tp92 can induce IL‐8 secretion in macrophages and HMEC‐1 cells.^17^ To avoid elimination by the inflammatory response of hosts, pathogens use various strategies to weaken the secretion of inflammatory factors by hosts or induce the hosts to secrete only cytokines beneficial for the pathogens. For example, the YopJ protein of *Yersinia pestis* inhibits the NF‐κB and MAPK inflammatory pathways to reduce the production of inflammatory factors.^62^ The *T. pallidum* antigen TpF1 promotes the secretion of the anti‐inflammatory factor IL‐10 to resist elimination by inflammation.^63^ Interestingly, further research showed that Tp92 failed to increase the TNF‐α, IL‐1β, IL‐6, IL‐10, IL‐18 and MCP‐1 levels and slightly elevated the IL‐8 levels via NF‐κB in THP‐1 cells. These data suggest that Tp92 recognizes CD14 and TLR2, transfers the signal to the downstream MyD88 pathway, and activates NF‐κB to mediate the production of IL‐8. Here, our data demonstrate that Tp92 induces cell death without aggravating inflammatory responses, possibly by inducing massive THP‐1 cell death such that the number of viable cells that can secrete inflammatory factors is sharply decreased. This scenario favours the survival of *T. pallidum* in the hosts and prevents the removal of *T. pallidum* by strong inflammatory responses. Meanwhile, Tp92 induces the production of IL‐8, which causes the accumulation of neutrophils and lymphocytes to the site of infection, increases the degree of infiltration of inflammatory cells in the infected area, and aggravates the immunopathological damage of the site.^64^ On the other hand, IL‐8 promotes the release of elastase by neutrophils, damages endothelial cells, and facilitates the transport of *T. pallidum* across the endothelial barrier and into the blood.^65^ The TpF1 protein induces the secretion of IL‐8 by hosts, promotes skin and mucosal vascular proliferation in patients with syphilis, increases vascular permeability, and helps the spread of *T. pallidum* throughout the body via the blood vessels. Simultaneously, vascular proliferation leads to the accumulation of nutrients and energy, providing material support for the survival of *T. pallidum*.^66^ Further study is required to determine whether IL‐8 secretion induced by Tp92 has a similar effect.

**Figure 12:**
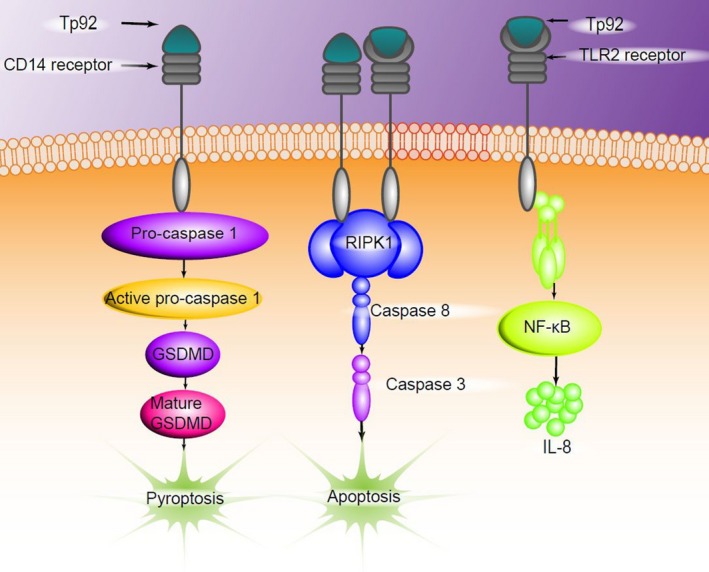
The outer membrane protein Tp92 of *Treponema pallidum* induces human mononuclear cell death and IL‐8 secretion

Our data regarding the effect of Tp92 on PBMCs showed that Tp92 induces the death of monocytes among PBMCs. However, the effects of this protein on T cells or dendritic cells are small, probably because monocytes, but not T cells or dendritic cells, exhibit abundant CD14 and TLR2 expression on their surfaces.^67^, ^68^ In conclusion, the present study demonstrates that the Tp92 protein induces the death of human mononuclear cells and the secretion of IL‐8 at the early stage of *T. pallidum* infection (Figure 12). This mechanism may help *T. pallidum* escape recognition and elimination by the innate immune response system of the host.

## CONFLICT OF INTEREST

All authors declare no financial competing interests. All authors declare no nonfinancial competing interests.
